# Effect of Lower Limb Muscle Fatigue on Fall Risk for Transfemoral Amputee: A Pilot Study

**DOI:** 10.1155/2021/4357473

**Published:** 2021-10-15

**Authors:** Mohd Khairuddin Mohd Safee, Noor Azuan Abu Osman

**Affiliations:** ^1^Department of Biomedical Engineering, Faculty of Engineering, University of Malaya, Kuala Lumpur 50603, Malaysia; ^2^Department of Science Rehabilitation, Faculty of Health Sciences, University Sultan Zainal Abidin, 21300 Kuala Nerus, Terengganu, Malaysia

## Abstract

Muscle fatigue is a decline in muscle maximum force during contraction and can influence the fall risk among people. This study is aimed at identifying the effect of fatigue on prospective fall risk in transfemoral amputees (TFA). Fourteen subjects were involved in this study with TFA (34.7 ± 8.1 yrs, *n* = 7) and normal subjects (31.1 ± 7.4 yrs, *n* = 7). Fatigue of lower limb muscles was induced with the fatigue protocol. Subjects were tested prefatigue and postfatigue using the standardized fall risk assessment. All results were calculated and compared between pre- and postfatigue to identify fatigue's effect on both groups of subjects. The results showed that the fall risk increased significantly during pre- and postfatigue for TFA (*p* = 0.018), while there were no significant differences in normal subjects (*p* = 0.149). Meanwhile, the fall risk between TFA and normal subjects for prefatigue (*p* = 0.082) and postfatigue (*p* = 0.084) also showed no significant differences. The percentage (%) of increased fall risk for TFA was 19.2% compared to normal subjects only 16.7%. However, 61.4% increased of % fall risk in TFA after fatigue by using the baseline of the normal subject as the normalized % of fall risk. The increasing fall risks for TFA after fatigue are three times higher than the potential fall risk in normal subjects. The result indicates that they need to perform more precautions while prolonging lower limb activities. These results showed the implications of fatigue that can increase the fall risk due to muscle fatigue from repetitive and prolonged activities. Therefore, rehabilitation programs can be done very safely and precisely so that therapists can pursue fitness without aggravating existing injuries.

## 1. Introduction

Fatigue has been generally described as a temporary decrease in performing physical activities and mental energy that the individual perceives [1]. Enoka and Duchateau [2] define fatigue as “a disabling symptom in which physical and cognitive function is limited by interactions between performance fatigability and perceived fatigability.” As a result, fatigue can affect body balance and have significant consequences, such as reduce the muscle force [3], slower choice reaction time [4], lower obstacle avoidance [5], reduce walking speed, increase overall fall, and reduce lower limb strength [6].

Frequently, fatigue is reported to have a few effects among older adults with and without disease and bonds with several fall risk factors, including slower gait speed, worse physical function, and greater disability [6]. Prolonged walking that can induce fatigue has been shown to increase step width variability and reduce the minimum footstep clearance, and these related factors indicate an increase in tripping risks [7]. Parijat Lockhart [3] mentioned that localized muscle fatigue could be a risk factor in causing slip-induced falls. There are still limited studies indicating that fatigue may increase fall risk [8], and none of them examines the future relationship between fatigue and fall risk in a transfemoral amputee.

The loss of a lower limb frequently requires longer rehabilitation sessions for a patient to return to a high level of daily function after receiving a prosthesis [9]. The majority of the population of amputee patients are commonly from elderly patients with vascular disease and diabetes [10]. Report from the Centers for Disease Control and Prevention, 28.7% of older people can fall within one year [11]. Meanwhile, 50% of amputees have falling experiences at least once a year, with 40.4% of these resulting in injury and rehospitalization [12]. However, the fall data for the active young amputee is still lacking. Moreover, it has been found that 56.1% of the lower limb amputees reported falling, and 26.8% reported fall-related injury [13]. The fall may result in joint dislocations, bone fractures, muscle torn, and sprains [14]. Therefore, rehabilitation for lower limb amputees should be more focused on preventing falls during walking or physical movement to improve everyday activities.

However, the effect of fatigue on prospective falls for transfemoral amputees has not yet been determined. The main idea is that old age, impaired postural stability, history of previous falls, and chronic disease burden are associated with increased fall risk [13, 15, 16]. In the meantime, rehabilitation's goal for a lower limb amputee is to perform activities of daily living independently by restoring their balance and functional mobility [17]. Walking and physical activities are beneficial for enhancing and maintaining functional mobility of the lower extremities, whether through physical rehabilitation or recurrent daily tasks [17]. However, repetitive and prolonged exercise will associate with muscular fatigue, disturb balance control, and increase the risk of falling during the activity itself, such as repetitive exercise [18].

The purpose of this study is to identify the effect of lower limb muscle fatigue on fall risk for TFA. This investigation examined the association between fatigue and fall risk in TFA after fatigue protocol. We hypothesized that TFA would have higher fatigue and an increased risk of falling than normal subjects with lower limb muscle fatigue. We assumed that the effects of fatigue induced by lower limb activity would increase the fall risk in TFA. Therefore, therapists need to identify the impact of muscle fatigue on transfemoral amputee patients with fall risk to prevent falling. Furthermore, rehabilitation programs can be done very safely and precisely so that therapists can pursue fitness without aggravating existing injuries. In addition, by knowing the effect of fatigue on TFA, the therapist will give more caution in handling TFA.

## 2. Materials and Methods

Fourteen participants were recruited to participate in this research. Seven of them were unilateral transfemoral amputation (TFA), and seven were normal subjects. TFA subjects were recruited from BioApps Prosthetics Orthotics Center, University of Malaya, based on the following criteria: (1) male subjects, (2) normal body mass index (BMI) range 18.5 kg/m^2^ to 24.9 kg/m^2^, (3) 18 to 50 years old and no history of peripheral vascular disease or neurological/musculoskeletal disorders, (4) 2 years or more experience with a definitive prosthesis, (5) able to walk unassisted and unaided more than 30 minutes, (6) Medicare Functional Classification Level 3 ambulator or higher status, and (7) score 5 grade for manual muscle testing (MMT) for lower limb muscles. These criteria were identified to reduce other factors that may contribute to other reasons for fatigue.

Meanwhile, the exclusion criteria for his research are (1) feel pain in their residual limbs, (2) cannot stand unsupported for more than 30 minutes, (3) had experience in research using the same types of equipment or protocol, and (4) uncomfortable with their prosthesis by scoring less than 7 for a rating on their prosthetic socket according to a comfortable scale. The comfortable scale was described by Hanspal et al. [19]: “0 – 10 scale where 0 and 10 represented the most uncomfortable and the most comfortable socket imaginable.” Approval from the Institutional Review Board for the test procedure was obtained from the University Medical Committee (MEC 895.7) and was run from 2018 till 2020. This research was registered in a WHO compliant trial registry (Thai Clinical Trials Registry: TCTR20210805001). This research's sample size is based on a representative sample, using the Krejcie and Morgan sample size technique [20]. The sample size in this study was based on the known population, which was recruited from the Bioapps Sdn. Bhd. at University of Malaya Medical Centre.

The subjects completed the sit-to-stand activity as part of the fatigue protocol (STS). This fatigue protocol was referred from the study by Bryanton and Bilodeau [21]: “subjects were seated on an armless bench (44 cm deep, 440 cm wide, 46 cm high) with subjects' feet were positioned shoulder width apart, barefoot, with heal and toe positions marked on the force platform to ensure that the feet did not move during the fatigue protocol. Prior to standing, subjects were instructed to sit in a comfortable erect posture with their arms folded across their chest to deter the use of compensatory arm swinging. For each STS repetition, subjects were instructed to stand up from the bench in a fully extended erect posture, stop briefly, and return to the initial seated position while maintaining foot contact with the force platforms at all times. STS repetitions were performed in time with the beat of an audible metronome set to 35 beats per minute (first beat signaled to ascend, the second beat signaled descent) until: (a) they could no longer maintain the given pace for five consecutive beats, (b) voluntary exhaustion occurred, or (c) 30 minute cut-off time was reached” [21].

The subjects were assessed fall risk using a computerized fall risk screening test, Biodex Balance System SD Inc., Shirley, NY (BBS). This system showed showed strong to excellent test-retest reliability for balance assessment [22] and may be clinically useful method to detect balance impairments [23]. The BBS's functions are to identify the fall risk and train neuromuscular control. The BBS software provided the fall risk index age-adjusted range. The BBS can assess the subjects' static and dynamic postural stability by recording their anteroposterior and mediolateral axes. The circular platform can move freely, and the subjects needed to maintain the vertical projection of their bodies. Meanwhile, they were instructed to maintain their center of gravity at the midpoint of the platform. A screen located in front of their face showed the mark representing the moving vertical projection that their body produced. Then, the subjects were asked to undergo three trials of 20 seconds each, starting with a platform setting of 6 and finishing with a platform setting of 2, with ten-second rest periods in between. The subjects were instructed to stand bilaterally with feet shoulder width apart in the center of the platform. The fall risk indexes were recorded and compared between before fatigue and after fatigue.

The data normality was tested using the Shapiro-Wilk test due to the small number of subjects. The normality test showed that the data was not normally distributed. We compared the fall risk index of TFA and normal subjects before and after fatigue using the Wilcoxon signed-rank test. Then, the results were also compared between TFA and normal subjects using the Mann–Whitney *U* test. The percentage (%) of fall risk in both groups was also calculated by normalizing the score % to the baseline score of prefatigue. All statistical analysis was performed using the statistical software SPSS26.0 (Version26, IBMCorp., Armonk, NY).

## 3. Results

Fourteen (*n* = 14) subjects consented to participate in the study; seven subjects had unilateral TFA, and seven normal subjects served as controls. They were given written informed consent and were explained about the study protocol before participating in this study. All of the normal subjects and TFA are male. The control group had no neuromusculoskeletal pathology or impairments in gait and balance. All of the normal subjects were right hand and leg dominant. Four of the TFA subjects had the dominant leg amputated and three nondominant legs. All seven subjects' amputation aetiology was trauma. They were instructed to perform experimental protocols, and the results were recorded. All fourteen subjects successfully finished the experiment. Details of the subjects are shown in Table 1.

**Figure 1:**
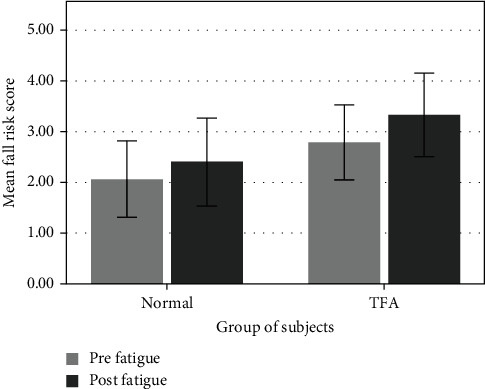
Mean fall risk score for normal and TFA subject group impact.

The data showed increases in fall risk scores for TFA and normal subjects after fatigue. The mean score of fall risk is showed in Figure 1. Initial analyses revealed different scores in both groups of subjects in the prefatigue. Consequently, the score result will be different in pre- and postfatigue due to different scores in the beginning. The scoring percentage (%) was determined by normalizing the prefatigue score data and calculating the increasing score % in both groups. The histogram showed a 19.2% increase in fall risk score for TFA; meanwhile, only 16.7% score increased for the normal subject group. These showed that fatigue could increase the potential of fall risk for all subjects. However, a significant difference was only found in the TFA group (*p* = 0.018), while normal subjects showed no significant difference in fall risk between pre- and postfatigue (*p* = 0.149).  Table 2 presents the statistical analysis for the fall risk test.

Each group showed an increase in fall risk after fatigue, but the TFA indicated a higher fall risk score after fatigue. The fall risk score for prefatigue was also higher in TFA (2.79 ± 0.79) than in the normal subjects (2.06 ± 0.81). The fall risk of TFA was already higher at the beginning before fatigue compared to the normal subject group. However, the statistical analysis showed that the fall risk score for TFA is no significant difference with normal subjects during both prefatigue (*p* = 0.082) and postfatigue (*p* = 0.084) (Table 3).

After fatigue, the TFA subjects demonstrated the high potential of fall risk with 61.4% increasing from the baseline of the normal subject group, while the normal subjects were increasing 16.7% from baseline. The result indicates that TFA has three times the risk of falling compared to the normal subject after fatigue.

## 4. Discussion

This study is aimed at assessing the fall risk on TFA and normal subjects during pre- and postfatigue. As expected, the results revealed that the fall risk increased in both groups of subjects, but the significant differences between pre- and postfatigue only showed on TFA. These results are consistent with the current literature, which also reported that fatigue would increase the fall risk [6, 8, 24]. However, most of the risk factors associated with falls were based primarily on elderly patient cohorts and not on amputees. Other studies also include other factors such as age-related, intellectual disability, lower limb strength, and cerebrovascular disease that are related to falling risk [25–27]. Our cohort population for TFA is younger than previously studied with an age range of 20 to 45 years old. Although our study recruited younger subjects, the results still showed an increase in fall risk. Thus, the age factor for TFA is not a primary factor of fall risk among TFA.

In our results, it has 61.4% increase in fall risk for TFA after fatigue. This result is aligned with previous studies that showed that 50% of lower limb amputees fall at least once a year, with 40.4 percent sustaining an injury [12]. However, the literature's population age cohort has an average age of 59 to 69 years and was only focused on the elderly. Previous studies have indicated that four years after amputation is related to increased risk [28]. At the same time, a younger sample demonstrated that amputees might be at a higher risk of falling and requiring rehospitalization during the first six months after amputation [12]. A few studies showed an important indication that amputation has a lower risk of fall with four years or greater having amputation [28], but the study still focused on elderly subjects. In our study, the subjects were below 50 years and only TFA subjects that Medicare Functional Classification Level 3 ambulator or higher. These criteria may reduce the contribution of other factors that will influence the result such as age-related factors.

This study did not assess the muscle potential related to the amputee's active and passive limbs. From previous studies, fatigue also can be induced through sustained contractions of a particular muscle group [29]. Therefore, an increase in fall risk can also be due to lower limb muscles' changed function for TFA. A TFA causes structural alterations in the muscles of the residual limb, as well as modifications in their functional role. For example, biceps femoris (BF) and rectus femoris (RF) have been transformed into uniarticular muscles that no longer function on the knee joint. Meanwhile, the adductor magnus (AM) loses its fixed insertion location, reducing the anchor point from which it can exert force. Aside from that, BF loses its connection to the fibula and tibia, RF loses its connection to the base of the patella, and AM loses the hamstring portion of its insertion to the femur's adductor tubercle. The example above showed the modification of muscles' role to adapt to the prosthetic device [30]. As a result, prosthesis wearers with unaffected lower limbs will develop a balanced strategy to compensate for the absence of the ankle joint and muscles resulting from amputation. Therefore, to maintain body alignment, lower limb amputees must adapt to the current situation and train to maintain balance while wearing a prosthesis [31].

Researchers have identified many conditions that contribute to falling and how to prevent falls. Exercise therapy is one of the procedures recommended for fall prevention, although by using the telecommunication medium or face to face in the rehab center or at home [32]. Another research showed that exercise is good for healthy people and patients [33] and would reduce the fall risk and increase the functional ability associated with disability [34]. Meanwhile, body composition, the proportion of fat and nonfat mass in the body, plays a significant role in balance and preventing falls [35]. A balanced diet and good body composition will increase physical fitness and improve body performance [36]. Continuing from this study, body composition and exercise therapy will have their role in maintaining body balance and reducing fall risk for TFA. However, previous studies also mention that repetitive and prolong physical activity can induce fatigue. So, therapists should have a proper exercise program and suggest healthy dietary intake to increase physical performance and reduce fall risk for TFA.

This research has several limitations. Due to specified restrictions, the number of TFA subjects in this study is limited, and the COVID-19 pandemic in early 2020 also hinders us from getting more subjects. Therefore, this study was underpowered with the *p* values only between 0.05 and 0.1 and indicated that the data might be a true effect. Although the results showed fall risk increased significantly in TFA compared to the normal subjects, many factors still need to identify the causes of increased fall risk in TFA, and these factors can be the limitation in this study. For example, body composition, exhaustion from cognitive and physical, age, body mass index, and type of prosthesis fatigue have their own impact on fatigue and fall risk [37–39]. Therefore, these factors may contribute to the inability to maintain postural control and may increase injurious fall risk. Finally, this study consisted of a small population and needed a more significant population with different criteria to identify the fall risk factors. Overall, this study showed that fatigue could be an important risk factor that may provide a novel avenue to identify appropriate interventions to lowering fall risk for TFA.

## 5. Conclusions

This study only assessed the short-term effects of performing fatiguing lower limb activity to identify falls risk for individuals above knee amputation. Therefore, for further research, we should identify the fall risk due to fatigue for other criteria of the amputee population such as gender differences, age, cognitive function, BMI differences category, nutrition factors, and body composition. These findings could have significant implications for assessing the risk of falling due to acute exercise-induced muscle exhaustion from repetitive multijoint movements, particularly in the lower limb muscles. This information can be utilized to educate TFA patients and urge the use of fall prevention interventions, particularly during rehabilitation and gait training. Therefore, rehabilitation programs can be done very safely and precisely so that therapists can pursue fitness without aggravating existing injuries.

## Figures and Tables

**Table 1:** General characteristics of subjects (mean ± SD).

	Control group (*n* = 7)	TFA group (*n* = 7)
Age (years)	31.1 ± 7.4	34.7 ± 8.1
Height (cm)	168.6 ± 2.7	167.1 ± 3.5
Weight (kg)	65.9 ± 5.3	63.6 ± 7.8
BMI (kg/cm^2^)	23.1 ± 1.8	22.7 ± 2.2

**Table 2:** Wilcoxon signed-rank test for normal subject and TFA during fall risk test.

Condition	Fall risk test	Median	Interquartile range	SD	*z*	*p*
Normal	Prefatigue	1.80	1.20	0.8142	-1.442	0.149
	Postfatigue	2.50	1.10	0.9416		
TFA	Prefatigue	2.70	1.70	0.7946	-2.366	0.018^∗^
	Postfatigue	4.10	1.20	1.3327		

^∗^ *p* < 0.05.

**Table 3:** Man-Whitney *U* test for pre- and postfatigue for normal subjects and TFA.

Fall risk	Group	Median	Interquartile range	SD	*z*	*p*
Prefatigue	TFA	2.70	1.70	0.79	-1.736	0.082
	Normal	1.80	1.20	0.81		
Postfatigue	TFA	3.20	1.00	0.89	-1.727	0.084
	Normal	2.50	1.10	0.94		

## Data Availability

The data are available on request through the author.
